# ASO Author Reflections: Guided by Fluorescence: Indocyanine Green Reverse Mapping and the Road Toward Lymphaticovenous Bypass in Axillary Surgery

**DOI:** 10.1245/s10434-025-17877-7

**Published:** 2025-07-19

**Authors:** Chu Luan Nguyen, Deepali Poels, Basilie Teoh, Pratik Rastogi, Jue Li Seah, Belinda Chan, Susannah Graham, Farhad Azimi, Cindy Mak, Carlo Pulitano, Sanjay Kumar Warrier

**Affiliations:** 1 Department of Breast Surgery, Chris O’Brien Lifehouse, Camperdown, NSW Australia; 2 Department of Surgery, Royal Prince Alfred Hospital, Camperdown, NSW Australia; 3 Department of Surgery, The University of Sydney, Camperdown, NSW Australia; 4 Department of Plastic and Reconstructive Surgery, Chris O’Brien Lifehouse, Camperdown, NSW Australia

## Past

Lymphedema is a chronic complication difficult to manage after axillary lymph node dissection (ALND) in breast cancer patients. Axillary reverse mapping (ARM) was developed to preserve upper limb lymphatics during ALND, aiming to reduce this risk. However, traditional ARM techniques using blue dye or radioisotopes have shown inconsistent detection rates and limited visualization.^1,2^ Additionally, concerns about oncologic safety, particularly the potential for ARM nodes to harbor metastases, have limited its clinical adoption.

## Present

The authors’ prospective trial evaluated the feasibility and oncologic implications of indocyanine green (ICG) fluorescence-guided ARM during ALND. They achieved a high ARM node detection rate of 95%, with real-time visualization of lymphatic pathways. However, 18.9% of the ARM nodes were metastatic, particularly in patients with larger tumors and higher nodal burden.^3^ These findings challenge the assumption that ARM nodes are consistently free of disease and suggest that routine preservation may be unsafe for patients with advanced axillary disease.

## Future

Future efforts should focus on refining patient selection for ARM node preservation. Tumor size and nodal stage may serve as useful predictors of ARM node metastasis. Additionally, combining ARM with immediate lymphatic reconstruction, such as lymphaticovenous anastomosis, may offer a safer strategy to reduce lymphedema risk without compromising oncologic outcomes.^4^ The ARM technique allowed for easy identification of the lateral lymphatic in the authors’ early series and also allowed for easier access to super-microsurgery anastomosis with confidence of the recipient lymphatic. This will be published by the authors in a follow-up trial.
